# Supplementary material for: Enhancers and genome conformation provide complex transcriptional control of a herpesviral gene
Source: Mol Syst Biol. 2024 Nov 19;21(1):2. doi: 10.1038/s44320-024-00075-0 (PMC11696879; doi:10.1038/s44320-024-00075-0)
Supplement: Supplementary file 16 — Expanded View Figures [file 44320_2024_75_MOESM16_ESM.pdf]

Expanded View Figures

Figure EV1. Supplementary screen data.

(**A**) Enrichment of individual guides at the ORF68 locus. Each dot represents a single guide, with the target location displayed on the x-axis and the average enrichment from two replicates on the y-axis. (**B**) Reproducibility of guide enrichments from two replicates. (**C–H**) Smoothed enrichment of guides at an indicated locus with annotated transcription start sites (Ye et al, 2019). For each guide, the median enrichment of a 100 bp window centered at the target locus was calculated along with an interquartile range (IQR) to represent the range of values. The median value is shown as a point, and IQR is shown as a shaded region. Regions significant ($p < 10^{-11}$) in sliding window analysis are shown in blue. NA indicates unannotated TSSs.

© The Author(s)

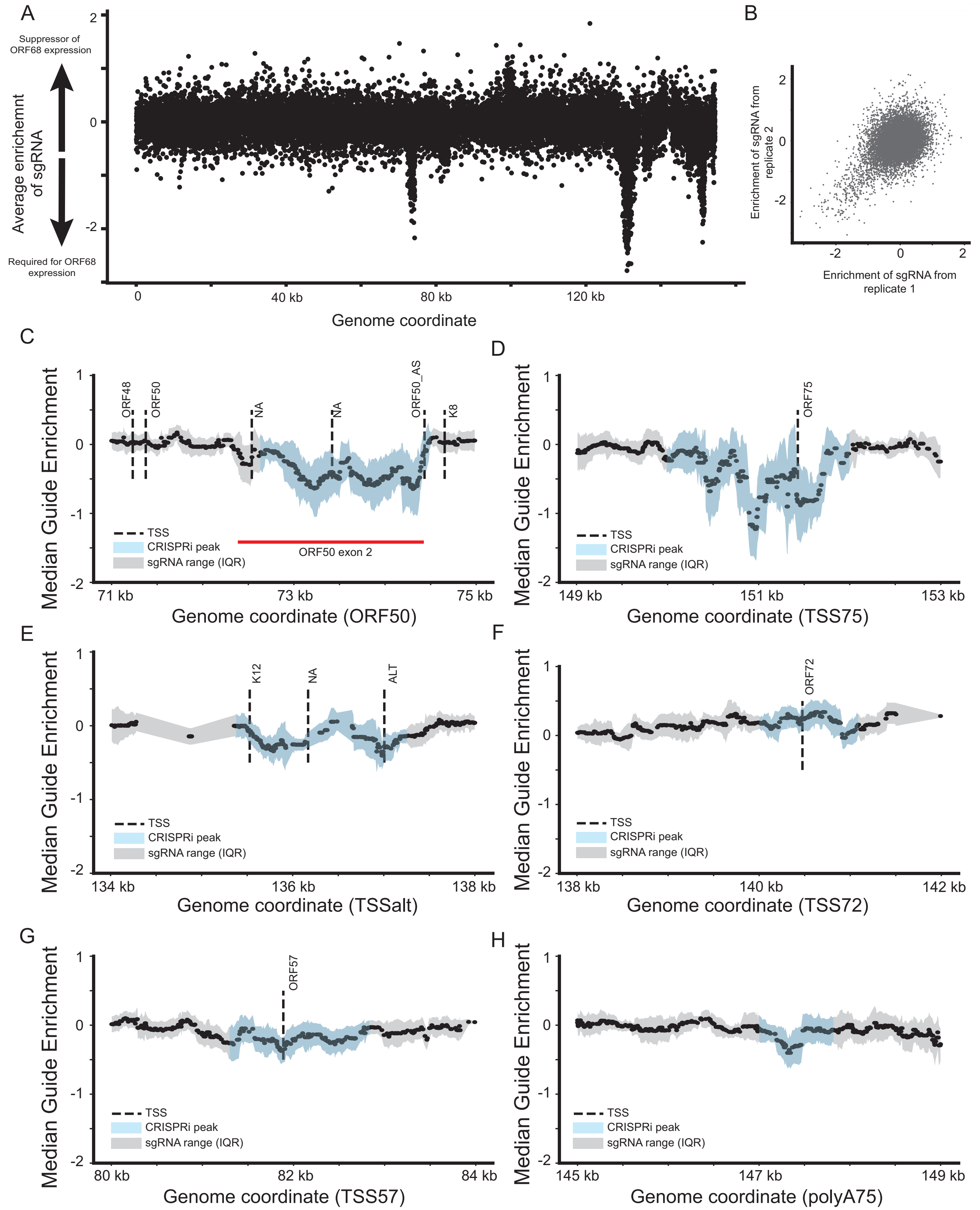
A
Suppressor of ORF68 expression
Required for ORF68 expression
Average enrichemnt of sgRNA
Genome coordinate
0
40 kb
80 kb
120 kb
B
Enrichment of sgRNA from replicate 2
Enrichment of sgRNA from replicate 1
C
Median Guide Enrichment
ORF48
ORF50
NA
NA
ORF50_AS
K8
TSS
CRISPRi peak
sgRNA range (IQR)
ORF50 exon 2
71 kb
73 kb
75 kb
Genome coordinate (ORF50)
D
ORF75
149 kb
151 kb
153 kb
Genome coordinate (TSS75)
E
K12
NA
ALT
134 kb
136 kb
138 kb
Genome coordinate (TSSalt)
F
ORF72
138 kb
140 kb
142 kb
Genome coordinate (TSS72)
G
ORF57
80 kb
82 kb
84 kb
Genome coordinate (TSS57)
H
145 kb
147 kb
149 kb
Genome coordinate (polyA75)

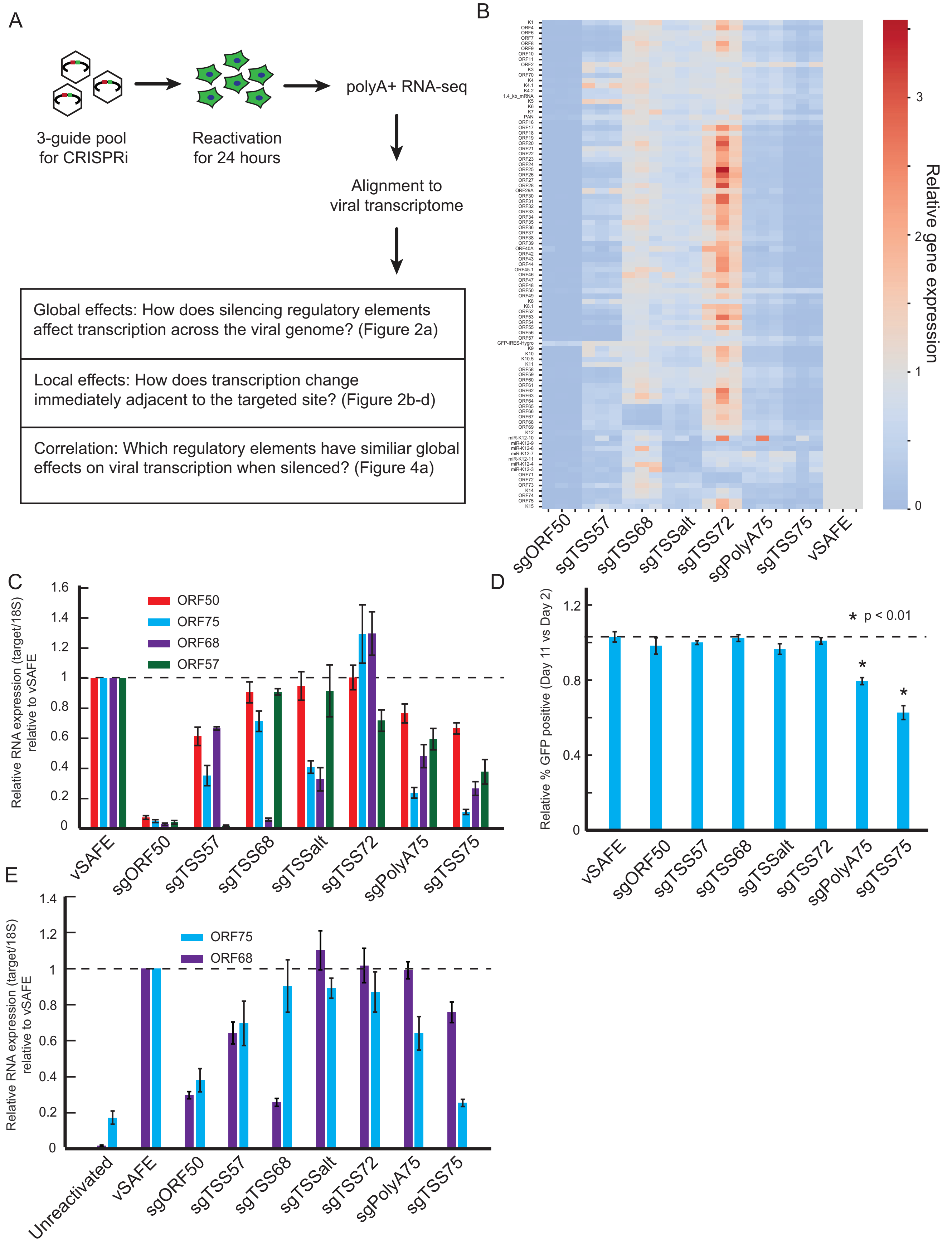
A
3-guide pool for CRISPRi
Reactivation for 24 hours
polyA+ RNA-seq
Alignment to viral transcriptome
Global effects: How does silencing regulatory elements affect transcription across the viral genome? (Figure 2a)
Local effects: How does transcription change immediately adjacent to the targeted site? (Figure 2b-d)
Correlation: Which regulatory elements have similiar global effects on viral transcription when silenced? (Figure 4a)
B
Relative gene expression
sgORF50
sgTSS57
sgTSS68
sgTSSalt
sgTSS72
sgPolyA75
sgTSS75
vSAFE
C
Relative RNA expression (target/18S) relative to vSAFE
ORF50
ORF75
ORF68
ORF57
D
Relative % GFP positive (Day 11 vs Day 2)
* p < 0.01
E
ORF75
ORF68
Unreactivated

Figure EV2. Supplementary for RNA-seq.

(**A**) Schematic showing set-up of RNA-seq experiment on CRISPRi cells infected with a three-guide pool, reactivated, and polyA+ RNA-seq at 24 h post-reactivation. (**B**) Heatmap indicating viral gene expression relative to the matched vSAFE replicate of each individual replicate. Rows are presented in genome order. Replicates from three independent reactivations. (**C**) RT-qPCR was used to measure how CRISPRi-based repression of the individual elements indicated on the x-axis influenced the levels of ORFs 50, 75, 68, and 57 mRNA. Error bars represent standard error centered on the mean of four independent reactivations. (**D**) Effect on latency measured by loss of virally encoded EGFP expression over 10 days. Mean values are presented relative to parental cells, and error bars are standard errors from three parallel replicates. *P* values are calculated by *t*-test; exact *p* values for marked values are 0.0031 for sgPolyA75 and 0.0013 for sgTSS75. (**E**) RT-qPCR of viral genes ORF75 and ORF68 in CRISPRi+ BCBL1 cells targeted with indicated guide RNAs at 24 h post-reactivation. Mean values are presented relative to 18S and vSAFE cells. Error bars are standard errors from five independent reactivations.

© The Author(s)

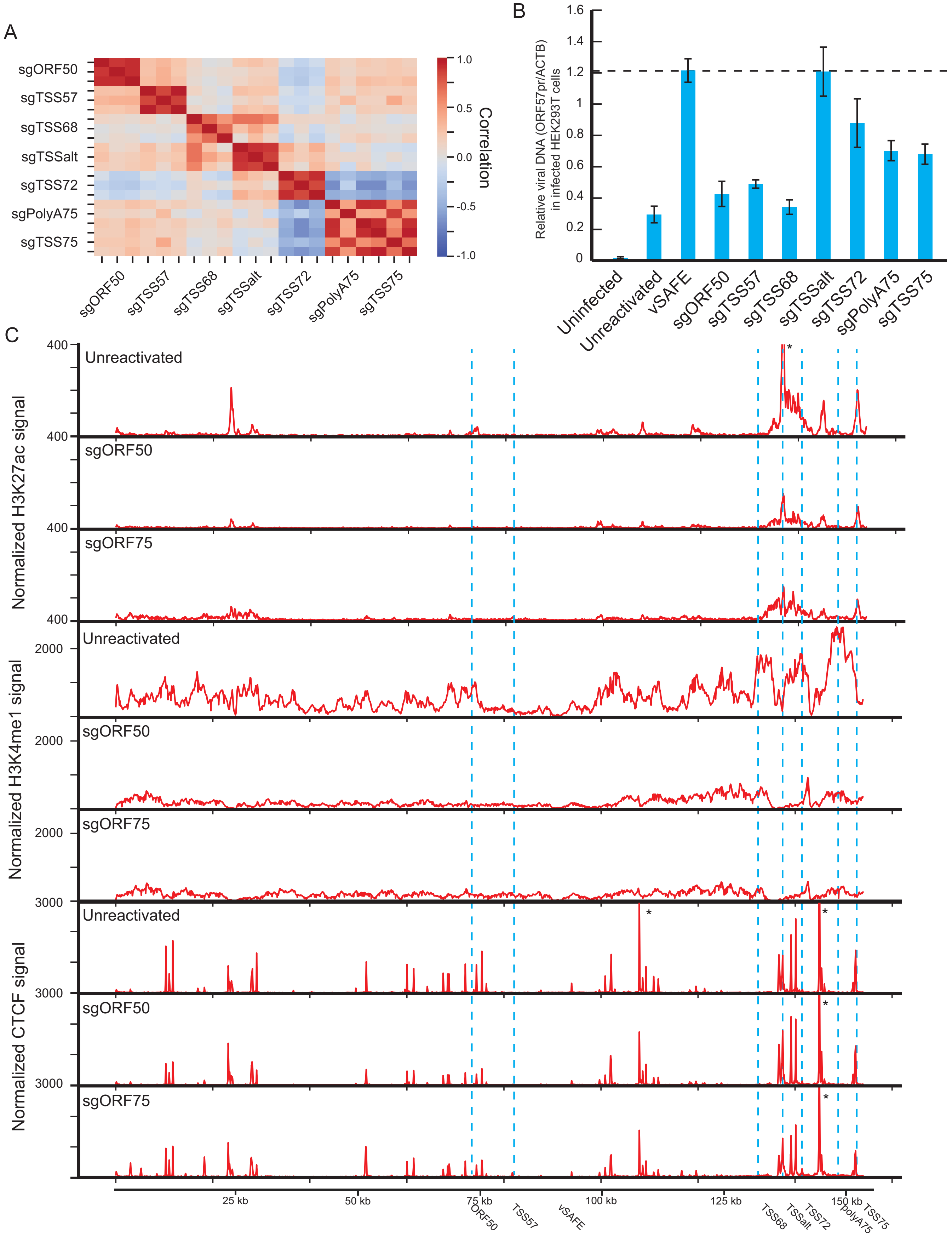
A
sgORF50
sgTSS57
sgTSS68
sgTSSalt
sgTSS72
sgPolyA75
sgTSS75
1.0
0.5
0.0
-0.5
-1.0
Correlation
B
Relative viral DNA (ORF57pr/ACTB) in infected HEK293T cells
1.6
1.4
1.2
1
0.8
0.6
0.4
0.2
0
Uninfected
Unreactivated
vSAFE
sgORF50
sgTSS57
sgTSS68
sgTSSalt
sgTSS72
sgPolyA75
sgTSS75
C
Normalized H3K27ac signal
Normalized H3K4me1 signal
Normalized CTCF signal
Unreactivated
sgORF50
sgORF75
400
2000
3000
25 kb
50 kb
75 kb
100 kb
125 kb
150 kb
ORF50
TSS57
vSAFE
TSS68
TSSalt
TSS72
polyA75
TSS75

 © The Author(s)

Figure EV3. Supplementary mapping data.

(**A**) Individual replicate correlation among RNA-seq. (**B**) Supernatant transfer assay measuring changes in KSHV virion production after knockdown of the indicated loci in CRISPRi+ BCBL1 cells. qPCR measurements of viral DNA content relative to host DNA content. Error bars represent standard error centered on the mean from four independent reactivations. (**C**) CUT&RUN signal from indicated mark in reactivated iSLK cells 24 h post-reactivation. Asterisks indicate peaks above the maximum signal graphed. The signal is averaged from three independent replicates.

© The Author(s)

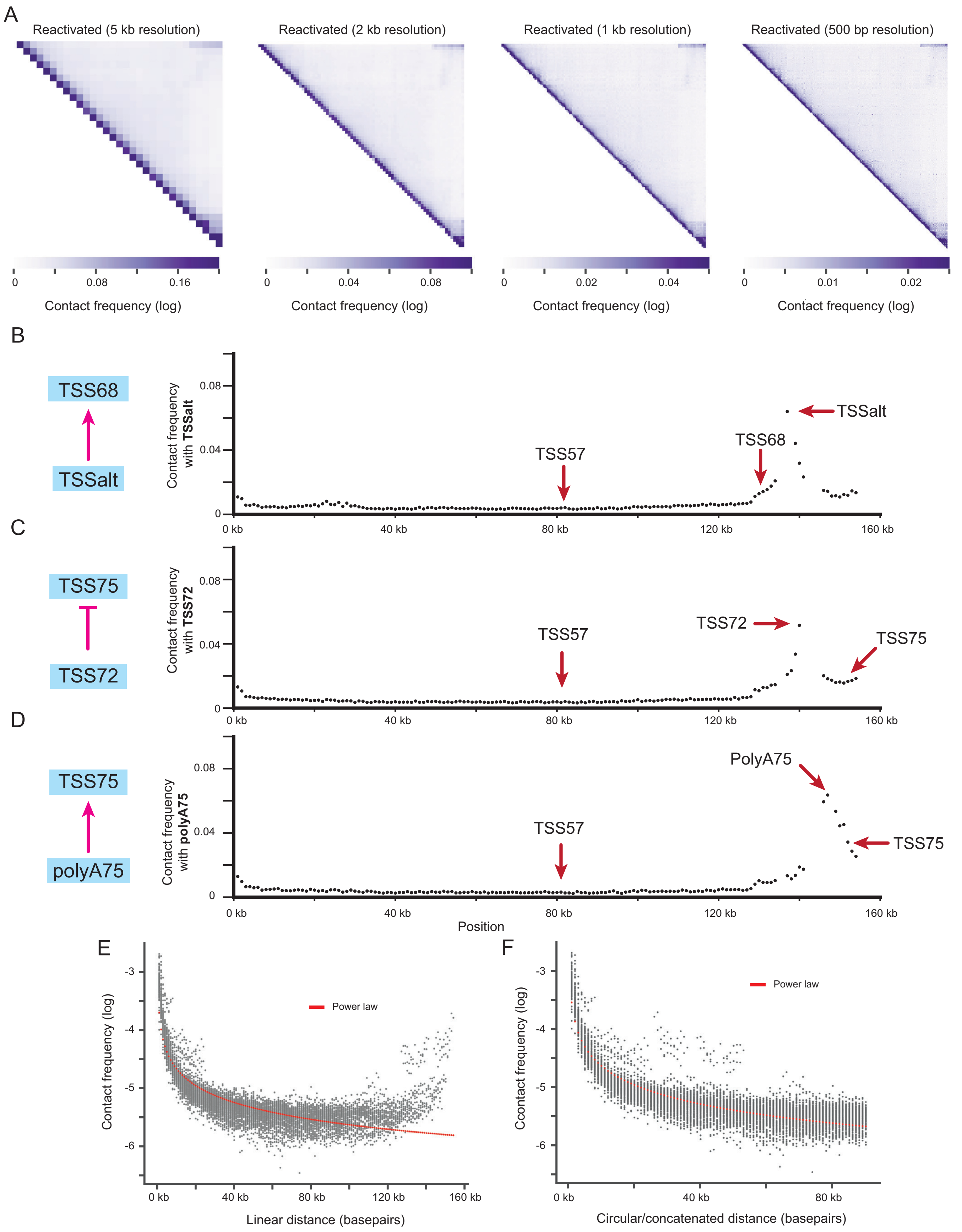
A
Reactivated (5 kb resolution)
Reactivated (2 kb resolution)
Reactivated (1 kb resolution)
Reactivated (500 bp resolution)
Contact frequency (log)
B
TSS68
TSSalt
Contact frequency with TSSalt
TSS57
C
TSS75
TSS72
Contact frequency with TSS72
D
polyA75
PolyA75
Contact frequency with polyA75
Position
E
Power law
Contact frequency (log)
Linear distance (basepairs)
F
Ccontact frequency (log)
Circular/concatenated distance (basepairs)

 © The Author(s)

Figure EV4. Hi-C data supplement.

(**A**) Contact frequency of reactivated sample at 5 kb, 2 kb, 1 kb, and 500 bp resolution. (**B–D**) Contact frequency between (**B**) TSSalt, (**C**) TSS72, and (**D**) polyA75 and other locations in the viral genome at 1 kb resolution. (**E**, **F**) The observed relationship between observed contact frequency and distance between regions when calculated using (**E**) a linear distance metric or (**F**) a circular/concatenated distance metric.

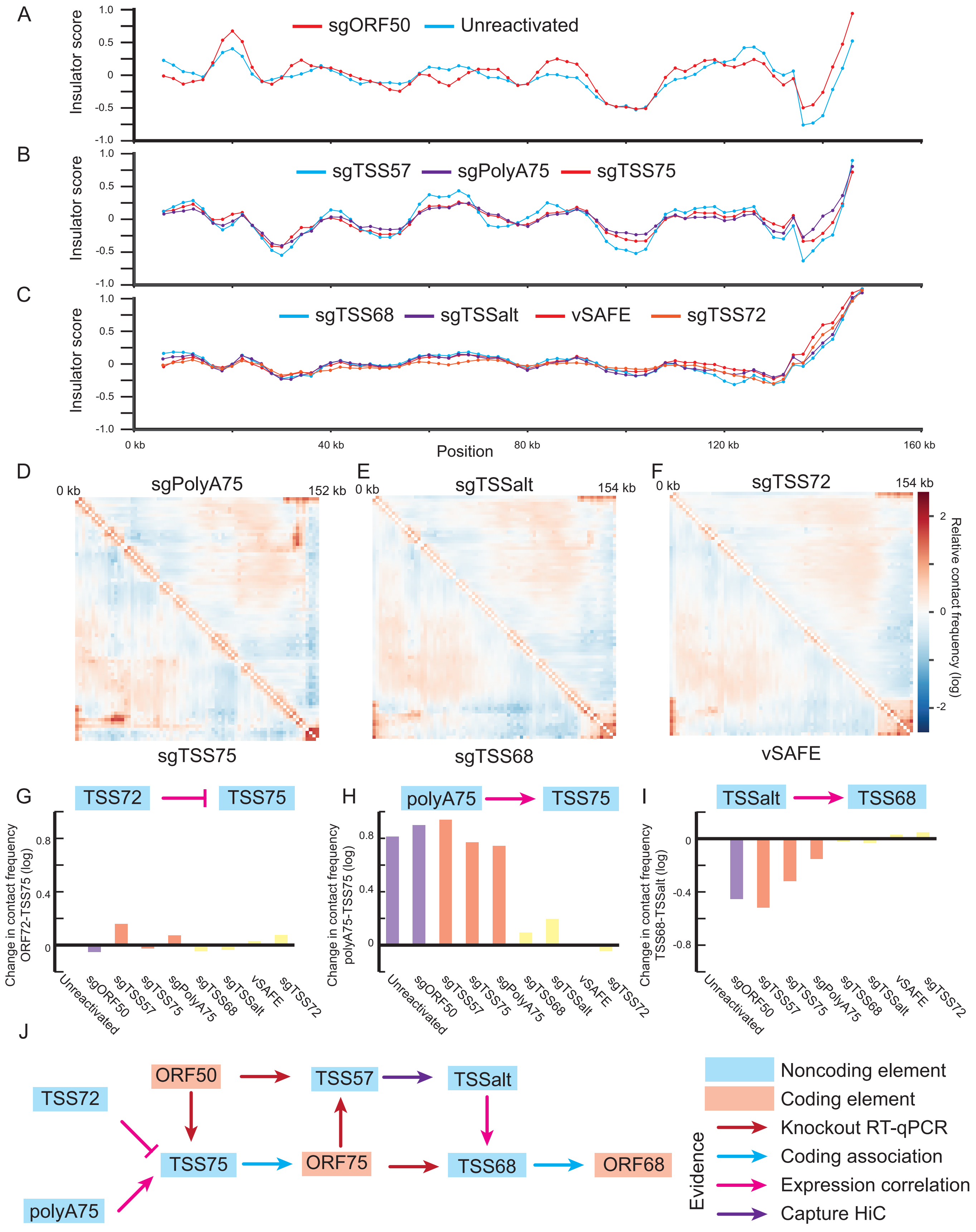
A
Insulator score
sgORF50
Unreactivated
B
Insulator score
sgTSS57
sgPolyA75
sgTSS75
C
Insulator score
sgTSS68
sgTSSalt
vSAFE
sgTSS72
0 kb
40 kb
80 kb
120 kb
160 kb
Position
D
sgPolyA75
0 kb
152 kb
sgTSS75
E
sgTSSalt
0 kb
154 kb
sgTSS68
F
sgTSS72
0 kb
154 kb
vSAFE
Relative contact frequency (log)
G
TSS72
TSS75
Change in contact frequency ORF72-TSS75 (log)
H
polyA75
TSS75
Change in contact frequency polyA75-TSS75 (log)
I
TSSalt
TSS68
Change in contact frequency TSS68-TSSalt (log)
Unreactivated
sgORF50
sgTSS57
sgTSS75
sgPolyA75
sgTSS68
sgTSSalt
vSAFE
sgTSS72
J
TSS72
ORF50
TSS57
TSSalt
TSS75
ORF75
TSS68
ORF68
polyA75
Noncoding element
Coding element
Evidence
Knockout RT-qPCR
Coding association
Expression correlation
Capture HiC

 © The Author(s)

Figure EV5. Structural changes supplement.

(**A–C**) Insulator scores for marked conditions measuring the relative frequency of reads crossing a given location. A more negative value indicates a strong insulator. Local minima were used to define the regions marked in Fig. 6E–G. (**D–F**) Relative contact frequency at 2 kb resolution for (**D**) sgPolyA75 and sgTSS75, (**E**) sgTSSalt and sgTSS68, and (**F**) sgTSS72 and vSAFE samples. Positive values indicate greater interaction than expected. (**G–I**) Change in contact frequency from reactivated cells between (**G**) TSS72 and TSS75, (**H**) polyA75 and TSS75, and (**I**) TSS68 and TSSalt at 2 kb resolution. (**J**) The final model for a regulatory circuit is based on all available data.

© The Author(s)
